# GIS-based intelligent planning approach of child-friendly pedestrian pathway to promote a child-friendly city

**DOI:** 10.1038/s41598-024-58712-5

**Published:** 2024-04-07

**Authors:** Kailun Fang, Suzana Ariff Azizan, Huiming Huang

**Affiliations:** 1 Guangzhou Urban Planning and Design Co., Ltd., Guangzhou, 510030 China; 2 Department of Science and Technology Studies, Faculty of Science, Universiti Malaya, 50603 Kuala Lumpur, Malaysia

**Keywords:** Child-friendly pedestrian pathway, Geographic information systems (GIS), Sustainable development, Smart city, Child-friendly city, Intelligent planning, Environmental social sciences, Health care, Engineering

## Abstract

Pedestrian safety, particularly for children, relies on well-designed pathways. Child-friendly pathways play a crucial role in safeguarding young pedestrians. Shared spaces accommodating both vehicles and walkers can bring benefits to pedestrians. However, active children playing near these pathways are prone to accidents. This research aims to develop an efficient method for planning child-friendly pedestrian pathways, taking into account community development and the specific needs of children. A mixed-methods approach was employed, utilizing the Datang community in Guangzhou, China, as a case study. This approach combined drawing techniques with GIS data analysis. Drawing methods were utilized to identify points of interest for children aged 2–6. The qualitative and quantitative fuzzy analytic hierarchy process assessed factors influencing pathway planning, assigning appropriate weights. The weighted superposition analysis method constructed a comprehensive cost grid, considering various community elements. To streamline the planning process, a GIS tool was developed based on the identified factors, resulting in a practical, child-friendly pedestrian pathway network. Results indicate that this method efficiently creates child-friendly pathways, ensuring optimal connectivity within the planned road network.

## Introduction

With 18 percent of China’s population under the age of 14, children constitute a significant demographic segment^1,2^. However, many Chinese metropolitan areas, characterized by expansive low-density urban landscapes and auto-dependent transit systems, lack adequate provisions for children. Recognizing this deficiency, there is an imperative to initiate child-friendly planning and research.

The concept of a child-friendly city, inspired by the UN Convention on the Rights of the Child, aligns seamlessly with Sustainable Development Goals (SDG) 3 and 16^3^. SDG3 addresses crucial child health issues, while SDG16 aims to create inclusive societies with equal access to justice. Ensuring children's well-being involves protection, a secure environment, and access to essentials, harmonizing with both SDG’s objectives^4,5^.

Children’s unique requirements are frequently disregarded in the setting of fast urban growth and high-rise constructions, which leaves a shortage of basic amenities and open spaces^6^. Smaller families and changing demographics underscore the need for adaptive infrastructure prioritizing children’s needs^7^.

Public health experts emphasize the pivotal role of social spaces, highlighting informal areas like pocket parks as popular playgrounds for children^8^. The quality of these spaces significantly influences children's well-being^9^. Access to play areas, independence, and parenting norms crucially determine children’s attraction to community play spaces. Establishing connections between these spaces is a key strategy for fostering children’s well-being^10^.

In historical contexts, pedestrian pathways were insufficient, posing risks to children sharing space with vehicles^11,12^. Existing standards often overlooked the specific needs of children, as design decisions were primarily adult-centric. Recognizing the importance of considering children's preferences in Child-Friendly Pedestrian Pathways (CFPP) planning is pivotal^13^. Traditional planning methods are deemed inefficient, prompting a shift towards exploring intelligent CFPP planning for more effective and previously overlooked insights^14,15^.

The research aims to analyze and synthesize the concept of a child-friendly environment and its application in existing literature, enhancing our understanding of socio-physical attributes and actors contributing to its realization. The study’s foundation lies in two research questions: (1) What socio-physical factors are crucial for implementing a child-friendly pedestrian pathway? (2) How can we intelligently plan a child-friendly pedestrian pathway?

The paper consists of three parts, with the second section summarizing recent scholarship on child-friendly cities, pathways, and intelligent planning. The methodology, including case studies and drawing methods, explores intelligent planning. The paper concludes with a summary of the CFPP plan and the broader field of childhood research.

## Literature review

### Child-friendly city (CFC)

The foundation of the Child-Friendly Cities (CFC)initiative is rooted in a renewed emphasis on the "right to the city," aligning seamlessly with the latest normative frameworks and action plans introduced by UN-Habitat and UNICEF, dedicated to upholding human rights^16^. UNICEF^17^ defines a CFC as “a city, town, municipality, or any system of local governance committed to fulfilling child rights as articulated in the UN Convention on the Rights of the Child (CRC). It is a city or municipality where the voices, needs, priorities, and rights of children are an integral part of public policies, programs, and decisions” (p.10).

“Child-Friendly Cities” (CFC) are pivotal for prioritizing children’s rights and needs in urban development. Originating in East Asia and the Pacific in 1999, the concept gained momentum amid rapid urbanization. Despite new potential, challenges like increased vulnerabilities for children, socio-economic concerns, and infrastructure difficulties arise. Ensuring cities meet the needs of all citizens, especially the young, becomes paramount^18^.

The CFC Index, as explained in the report, is anchored in four fundamental principles derived from the ‘Convention on the Rights of the Child’^17^. These principles focus on equity, education, health and well-being, and security and protection, intricately interwoven with communal participation^17^. The guiding principles for building a child-friendly city include non-discrimination, making the best interests of children a primary consideration, recognizing the right to life, survival, and development, and respecting the views of the child^18^.

The development of child-friendly cities requires meticulous consideration of various parameters^9^, each playing a pivotal role in the planning and establishment of an environment conducive to children. These parameters cover safety, green spaces, diverse activities, independent mobility, social interaction, and involving children in decision-making^9,19^. Enhancing the physical environment aligns with meeting children's rights, involving creating and maintaining parks, play spaces, and children's services.

In a recent study, researchers identified four key features characterizing a child-friendly city were identified: (1) The presence of 'green lungs’; (2) Opportunities for creative and challenging play; (3) Spaces suitable for the entire family; and (4) Safe playgrounds and walking routes^20^. This research underscores the significance of these specific features in shaping urban environments that cater to the diverse needs and overall well-being of children.

A child-friendly environment ensures safety, access to services, cleanliness, and opportunities for play and learning. It promotes independent mobility, emphasizing overall growth and well-being^17,19,21^.

Crucial to determining the child-friendliness of a built environment is children's independent mobility^22–24^. Children residing in urban areas often experience growing limitations on their independent spatial mobility. These restrictions stem, in part, from safety concerns associated with traffic designed to accommodate the mobility needs of other citizens^25^. As the challenge of diminishing independent mobility for children in urban areas becomes apparent, addressing safety concerns comprehensively becomes paramount. Children’s restrictions underscore the need for proactive measures, shaping the built environment to support their autonomy. Urban planners and policymakers play a pivotal role in prioritizing children's needs and contributing to their well-being.

To enhance child-friendly urban spaces, a specific avenue is the focus on Child-Friendly Pedestrian Pathways (CFPP). Addressing challenges in children's navigation, CFPPs offer safe routes, actively encouraging autonomy and engagement with the urban landscape. This shift aligns with the broader goal of fostering urban spaces that comprehensively cater to the well-being and developmental needs of children.

### Child-friendly pedestrian pathway (CFPP)

Child-Friendly Pedestrian Pathways (CFPP) are purpose-built walkways exclusively designed for children, with a primary focus on meeting their needs and ensuring safety. Despite the perceived safety of pedestrian pathways for adults, persistent parental concerns regarding their children's safety highlight the paramount goal of CFPP—ensuring safety^26^. Additionally, CFPP strives to align with children’s interests, offering both formal and informal play environments by differentiating between structured and unstructured play. This approach creates a comprehensive and engaging pedestrian pathway system that is tailored specifically to children's needs, fostering a sense of security, and promoting active lifestyles^27^.

Pedestrian pathways, often perceived as functional conduits for movement, undergo a transformative shift in the context of child-friendly urban planning. CFPPs are envisioned to transcend mere functionality, evolving into vibrant spaces for play and exploration. This transformation includes the incorporation of playful elements such as interactive installations, colourful markers, and designated play zones strategically integrated along the pathways^28^. According to Makalew et al.^28^ the design of child-friendly pathways should ensure a safe surface for children's activities, considering their environment and movement patterns, and be effective and efficient in school and housing areas. These additions not only serve to promote physical activity but also play a crucial role in stimulating cognitive and social development among children. This guiding principle seamlessly translates into the strategic placement of child-friendly pedestrian paths within the urban fabric. These pathways are envisioned to traverse areas that genuinely capture children's attention, connecting key locations such as parks, schools, and cultural spaces. By establishing an interconnected network of pathways, the urban environment becomes not only traversable but also inherently attractive to children, fostering a sense of community and enhancing their overall urban experience.

Building on the idea of empowerment through participation, the design and use of child-friendly pedestrian pathways transcend the realm of mere physical infrastructures. These pathways become powerful tools for empowering children by providing safe, accessible routes that significantly contribute to their overall well-being. Through active involvement in the planning process, children develop a profound sense of ownership and agency in shaping their urban surroundings. This empowerment extends to their use of the pathways, fostering independence, confidence, and a positive connection to their environment.

The general principles outlined in the broader discussion about child-friendly cities find a specific and tangible application in the design and implementation of child-friendly pedestrian paths. By transforming pathways into playful, strategically located, and collaboratively designed spaces, urban planners can create environments that prioritize children's needs, fostering a sense of community, empowerment, and well-being. In doing so, cities not only become more accessible for their youngest residents but also set a precedent for inclusive and sustainable urban development.

### Child participation in planning

Creating a child-friendly city involves community engagement, urban planning, and effective policymaking, relying on a clear understanding of factors promoting children's well-being and development.

Urban planning assesses city design for children’s needs, ensuring accessible playgrounds, parks, and safe pedestrian pathways. An integral aspect of child-friendly urban planning revolves around the careful selection of engaging locations that align with children’s interests, ensuring they are safe, healthy, comfortable, and convenient^29,30^. To truly capture the essence of a child-friendly city, it requires public spaces equipped with appealing facilities for children to play, including specific sites that, although not explicitly identified by children, manifest as their own^31,32^. Prior research often neglects choosing places that truly interest children—a critical oversight in creating child-friendly cities. Identifying engaging locations, like parks and playgrounds, is crucial for effective planning.

At the heart of child-friendly urban planning lies an interactive process that actively incorporates the perspectives and voices of children. Collaborative methodologies^33–35^, including workshops, interactive sessions, and participatory design activities, emerge as essential tools for ensuring that cities are not only safe and functional but also resonate with the preferences and needs of their inhabitants. Research strongly suggests that involving children in the planning process is crucial for understanding their perspectives and empowering them to influence the shaping of their neighborhoods and cities^33^. Simultaneously, this approach guarantees that cities authentically reflect the identity of the community they serve.

Identifying the most optimal method for involving children in the community design process is critical. Six suggested methodologies involve research with young children: (1) multi-method and the Mosaic approach; (2) observation and ethnography; (3) language-based methods; (4) visual method; (5) creative and playful methods; and (6) children as co-researchers^36–38^. These methods involve children in planning, ensuring their perspectives shape child-friendly urban development. Participation in decision-making and urban planning involves adults and children, prioritizing well-being and ensuring inclusive, sustainable development.

### Urban planning theories: shaping cities through time

Urban planning evolves through diverse theories to understand and enhance urban dynamics. Tracing back to historical movements in the mid-nineteenth century, these aim to bring order to the chaos of industrial towns^39^. The late twentieth century witnessed the introduction of the phrase “sustainable development,” encapsulating the vision of an ideal societal state^40^. This era marked the inception of a paradigm shift towards sustainable building practices, gaining popularity for their environmentally beneficial attributes, including the use of renewable resources and materials^41^.

Urban planning theories, diverse in ideas, shape city development over time. Classifiable into pre-history, foundation years, modernism, post-modernism, and current eras, they signify distinct phases in the field’s evolution^42^. Each phase mirrors prevailing ideological challenges, shaping the overall evolution of urban planning.

Pre-history planned cities around social, cultural, and religious hierarchies. Early urban planning, influenced by anarchist movements, introduced concepts like Garden City, Radiant City, and Broadacre^43^. In the modernist era, emphasis on efficiency prioritized optimized land use through zoning and transportation^42^. Post-modernism in urban planning shifted from practice to academia, challenging practical links. Suburbanization caused a political economy shift as the middle class left the city core, impacting the historical urban fabric^42^. The current era emphasizes connected cities with transit-oriented development and environmental conservation^42^.

Urban planning evolves for better cities, integrating diverse strategies from industrialization to sustainability. Historical theories inform intelligent planning, utilizing advanced tech for adaptive, inclusive urban environments.

### Intelligent planning

In child-friendly city planning, intelligent planning, driven by technologies like AI, brings a promising paradigm shift. Traditional methods, well-intentioned but resource-consuming, can be made more efficient through AI integration, opening innovative avenues for child-friendly cities.

AI's capabilities extend to improving transportation efficiency and safety, optimizing electricity and water services, and reducing construction costs and unplanned overruns in the urban environment^43^. Using this technology, child-friendly city planning gains efficiency and streamlined processes. Intelligent planning methods, focusing on technology and data-driven insights, provide a more efficient alternative compared to traditional approaches.

As urban areas continue to evolve, the need for child-friendly spaces becomes increasingly crucial. Incorporating AI into the planning process allows for a more nuanced understanding of children's needs, preferences, and requirements^32,44^. AI can analyze vast amounts of data to identify optimal locations for playgrounds, safe pedestrian pathways, and other child-centric spaces^45^. The technology's ability to process and interpret information quickly contributes to creating environments that are not only functional but also tailored to the specific needs of young residents^16,45^.

Intelligent planning, like GIS-based smart transportation systems, enhances efficiency and safety in child-friendly city planning. These systems prioritize safety and convenience, fostering an environment for children's independent mobility^46^.

Despite these advancements, there exists a critical gap in integrating professional planning knowledge into the analysis of people's needs in GIS-based intelligent planning, as previous research often overlooked the expertise of professional planners^47,48^. To bridge this gap, a comprehensive approach that incorporates professional planning knowledge is crucial for child-friendly city planning. This integration ensures that the unique needs of children and communities are not only considered but deeply understood, leading to more effective and responsive planning strategies^49,50^.

AI and intelligent planning transform child-friendly city planning, prioritizing children's well-being and shaping technologically advanced, inclusive cities for the next generation.

## Methodology

All methods were carried out in accordance with relevant guidelines and regulations. The drawing research was approved by the Ethics Committee of Guangzhou Urban Planning and Design Co. Ltd. Informed consent was obtained from the legal guardian in Guangzhou, China.

### Case study method

**Figure 1:**
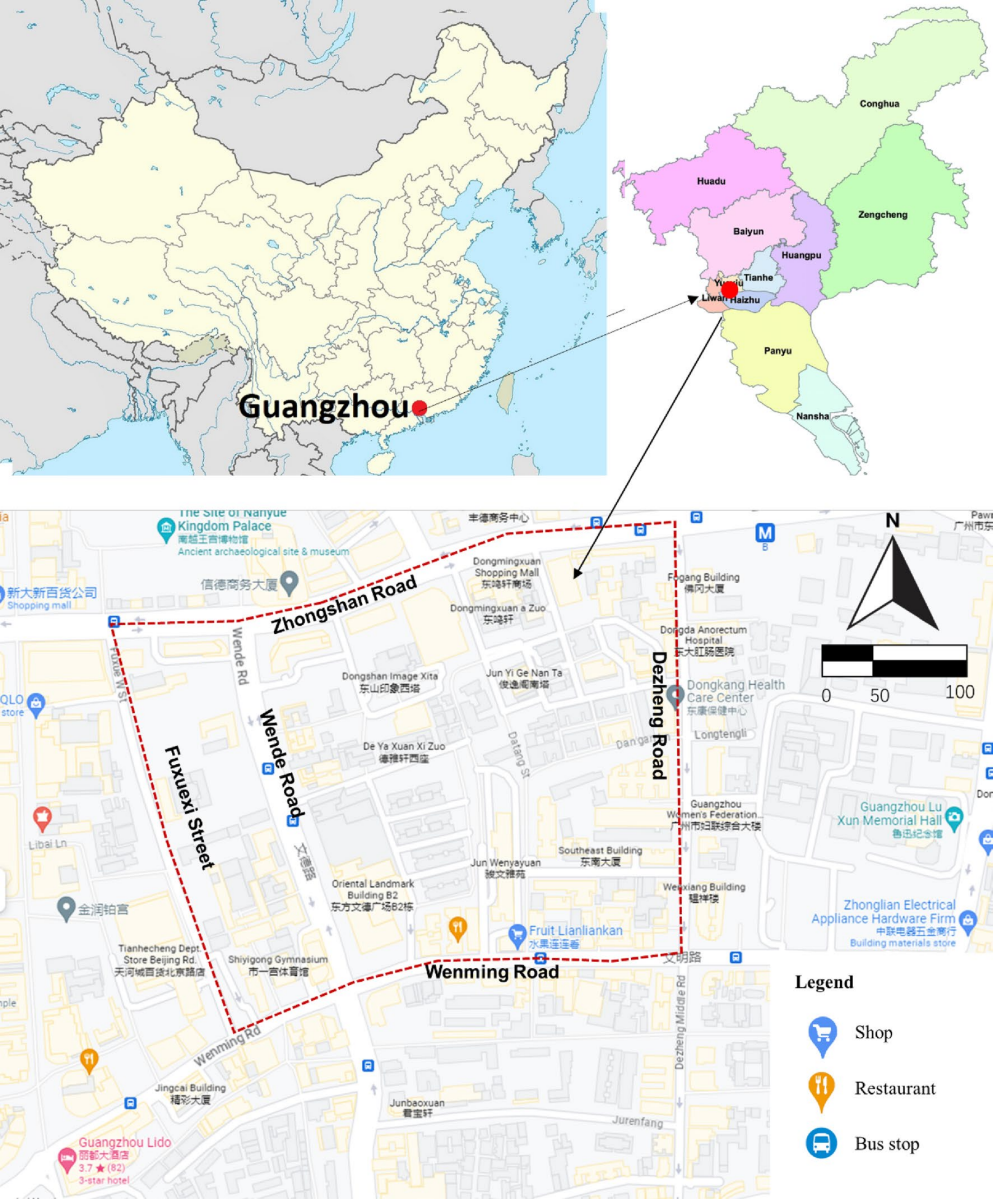
The Location of Datang Communality (Drawn by author).

The research selected the Datang Community as a case, which is located north of Guangzhou, China. Guangzhou is a major economic hub in South China, which is the second city to promote child-friendly cities (See Fig. 1).

This tool focused on the Datang case for two main reasons: First, Guangzhou aimed for urban sustainability through child-friendly city guidelines, with the community as the basic unit. Datang, a 38 ha child-friendly community project, was chosen for its emphasis on childcare, pedestrian pathways, and playgrounds. Second, the community's role in child development, highlighted in various studies, underscored the importance of the built environment, particularly pedestrian pathways, in facilitating social interaction, recreation, play, and learning.

### Drawing method

Children's drawings offer a glimpse into their thoughts and emotions, serving as a reflection of their inner world. As children are often naturally shy and find it challenging to express themselves verbally, drawing tests provide a quick, easy, and enjoyable method for them to communicate. Analyzing children's drawings had long been regarded as a systematic approach to evaluating their perceptions and experiences of the surrounding environment. Numerous studies have shown that children's drawings can provide indicators of various issues and potential solutions.

This study selected ‘Drawing’ as a tool for collecting primary data with 50 children, aged 2–6 years on their favorite spaces had been used by means of cluster random sampling method. The drawings were held in the kindergarten and daycare in the community. Through drawing, children were able to organize their internal thoughts and emotions and delivered clearer and more detailed narratives. This age group of children was selected as they can express their thoughts and opinions through drawings, but it needed to be further communicated with them about what they want to express, and the parents had done this with children. Additionally, they possessed a wish for their drawings to accurately portray real images of pictures or photographs.

The children were given instructions to draw places that interested them and where they desired to play. Prior to starting the drawing activity, they were provided with an explanation of the research’s objective. They were also informed about the materials they could use for the exercise. To facilitate computer scanning, the children were asked to draw on one paper. They were given the option to create black-and-white or color drawings. The children were given complete freedom to express themselves through their drawings. Oral consent for participation was obtained from the children, and they were informed that they could withdraw from the study at any time and that their identities would be kept confidential. Permission was also obtained from their teachers and parents.

### Survey method

Questionnaires were held to the kindergarten about the suggestions of the CFPP, the sample was 30. The sample aged from 3–4 was 44% and 4–6 was 56%. Girls were 45%, boys were 65%. Based on the correlation analysis, it was observed that children between the ages of 2 and 4 exhibited a higher tendency to visit green spaces, whereas those aged 4 to 6 showed a greater interest in educational resources. The preferences for other places were relatively consistent across different age groups.

### Data analysis method

#### Define criteria

**Figure 2:**
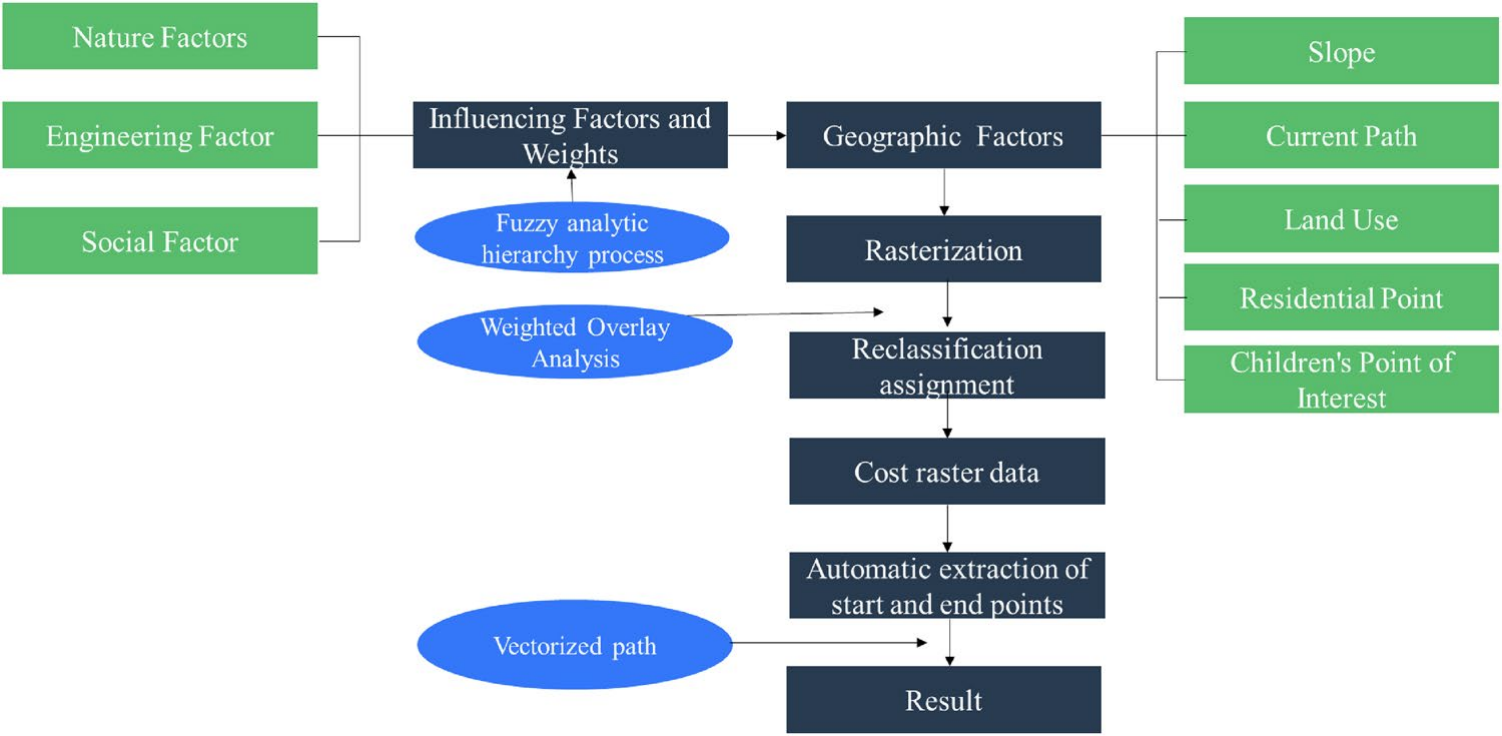
Framework of Data Analysis Method (Drawn by author).

Examine the essential considerations when formulating a pedestrian pathway plan that was specifically designed to be child-friendly. The criteria encompass various factors, such as the proximity of the pathway to schools, the incorporation of safety features, the availability and quality of sidewalks, the evaluation of traffic volume, adherence to appropriate speed the limits, presence of well-marked crosswalks, and convenient access to nearby parks or recreational areas. By taking these aspects into account, a comprehensive and well-rounded CFPP plan can be developed (See Fig. 2).

#### Data preparation

To adequately prepare the collected data for comprehensive analysis, it was essential to undertake the necessary steps before importing it into GIS software. By ensuring that all data was in a compatible format, it can seamlessly integrate with the chosen GIS software. Additionally, to facilitate meaningful spatial analysis, it was crucial to project the data onto a common coordinate system. This ensures that the spatial relationships and measurements within the data were accurately represented. By meticulously tending to these details, the data can be effectively utilized within the GIS software, enabling robust analysis and informed decision-making.

#### Weighting and prioritization

Assign weights to different criteria based on their relative importance. The fuzzy analytic hierarchy process (FAHP) was used to determine the influencing factors and weights of child pedestrian-friendly pathway planning, which were divided into four steps: (1) a hierarchical structure model was constructed to sort out the influencing factors and their relationships of community road planning; (2) the evaluation index was improved by a triangular fuzzy number. The fuzzy judgment matrix was constructed by pairwise comparison of rows. (3) checking the consistency of the fuzzy judgment matrix, and adjusting the fuzzy judgment matrix if it was inconsistent with the consistency; (4) calculating the weight of each index. To utilize the suitability modeling capabilities of GIS software to generate a composite score for each pathway segment, indicating its suitability as a CFPP. Prioritizing segments with higher scores for further consideration in the pathway plan.

#### Data analysis

Utilized network analysis tools to identify the most suitable routes for CFPPs, considering the defined criteria. Weighted overlay analysis was an important GIS spatial analysis that could assign weights to different criteria or factors and combine them to generate a final output map that represents the overall suitability or desirability of a location for a specific purpose. The calculation model was as follows.

1 $$S = \sum\limits_{i = 1}^{n} {F_{i} W_{i} \left( {i = 1,2 \cdots n} \right)}$$

where: S was the multi-factor comprehensive cost; Fi is the grid value of a single influencing factor; Wi is the weight corresponding to a single influence factor. n was the number of influencing factors involved in the cost calculation.

#### Visualization and mapping

To generate visual depictions or representations of the plan for the child-friendly pedestrian pathway. It suggests the creation of visual materials that illustrate how the pathway will be designed, its layout, and other relevant aspects. These visual representations could take the form of maps, diagrams, or conceptual illustrations that help communicate the intended design and features of the child-friendly pedestrian pathway (See Fig. 2).

## Result

### Children's point of interest (CPOI)

In the results, between the ages of 2 and 4, as children engage in scribbling activities, discernible shapes resembling cars or houses may begin to emerge in their drawings. When children reach the age of 4–6, their drawings tend to include simple elements like faces, stick figures, cars, trucks, trees, and houses. Understanding the specific areas of interest can be gleaned by examining the emotions conveyed through their drawings. For instance, bold and closely spaced strokes may indicate stress, strong emotions, determination, or anger, while softer marks suggest a more gentle disposition. The quality of lines drawn also holds significance; a figure created with light, wavering, and broken lines may suggest a hesitant and insecure child who is formulating thoughts as they go, whereas bold, continuous, and freely drawn lines express self-confidence and a sense of security.

**Figure 3:**
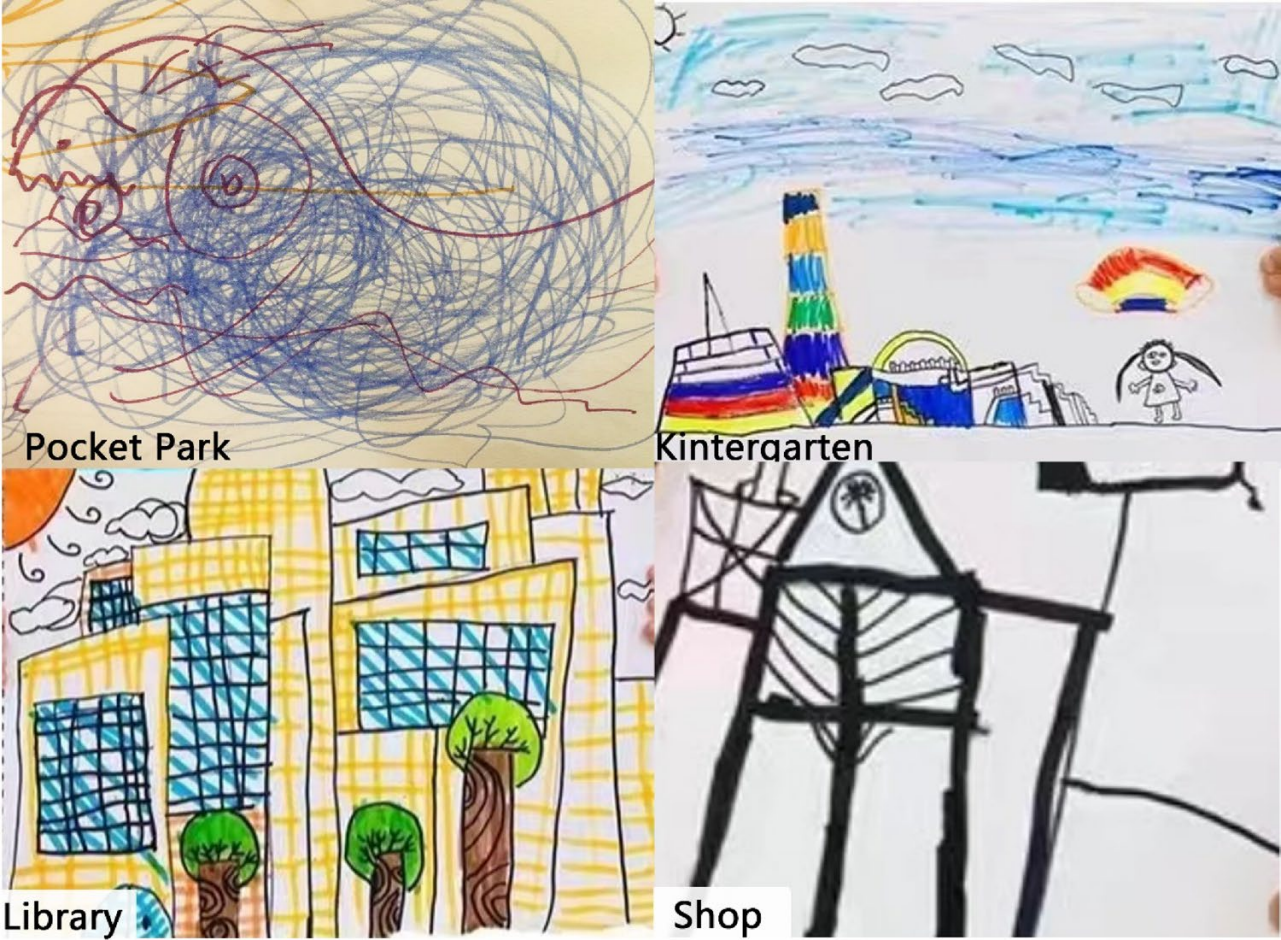
Examples of Children’s Painting.

**Figure 4:**
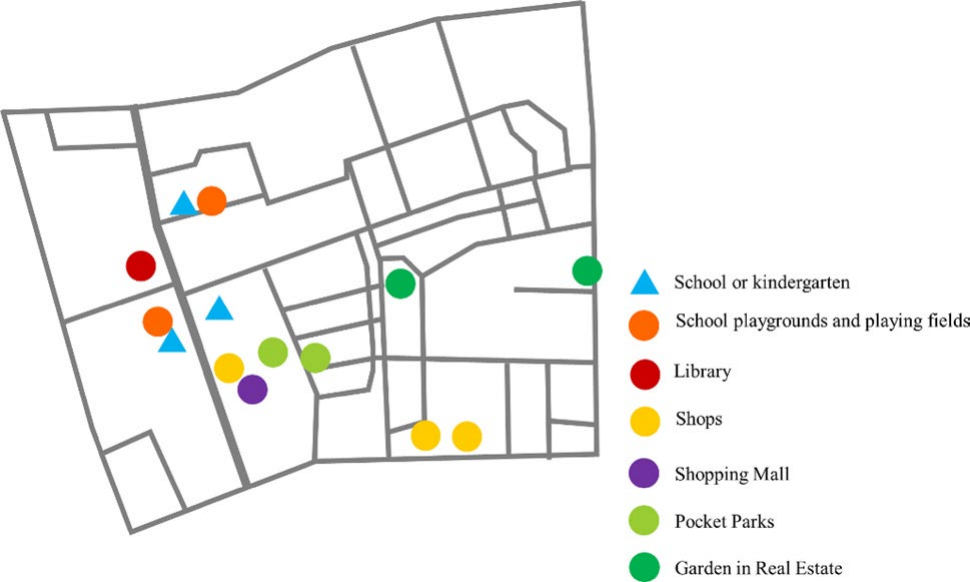
Map of CPOI.

The adjacent neighborhoods surrounding the school boasted a plethora of well-designed and accessible public spaces that held immense potential for children to actively partake in play and recreational endeavors. These carefully curated areas serve as enticing hubs for young individuals to unleash their energy, engage in physical activities, and enjoy leisurely pursuits, fostering a vibrant atmosphere of health and well-being. Children expressed a strong preference for outdoor spaces that were roomy, well-maintained, and had attractive landscaping. They emphasized the importance of cleanliness and the promotion of good health in these areas. Additionally, their artwork and designs frequently highlighted the significance of green, open spaces for engaging in various outdoor activities. Figure 3 demonstrates the types of places that children are interested in and use for active play, including public spaces such as school playgrounds and playing fields, and a large library. However, they were more likely to choose a mini-public space to play in, like the pocket park (small urban green space for recreation and relaxation in dense areas) with a pool, fish, or slides. In addition, the children often went to private spaces for play, including the shopping mall, where they travel with their family to eat and play on the indoor playground, and the private garden inside the neighborhood. And some interesting shops were also the main attractions, like pet shops or flower shops. Some children told parents they liked to visit the pet cat in one shop every day because their moms did not allow them to have pets (See Fig. 4).

### Data analysis in datang community

Pedestrians who rely on active transportation required adequate facilities, including child pedestrian pathways. Children, as pedestrians, were vulnerable to accidents due to their active movement and tendency to play while walking along the pedestrian pathway. Their unpredictable movements resulted in collisions with other users, such as pedestrians, cyclists, motorcyclists, and cars. When designing and planning a prototype for a child pedestrian pathway, it was important to consider the active movement of children and the objects they may use for play. Proper design and construction were crucial for ensuring the safety of child pedestrians in areas with high pedestrian and street user traffic. Therefore, the data selection focused on this specific target. The specific data processing processes for each type were as follows:

#### Define criteria

The assessment criteria tool for pedestrian assessment included three dimensions: (1) educational resources; (2) safety; and (3) play and recreation^51^. This study incorporated collinearity analysis for each dimension. In the initial dimension, the Variance Inflation Factor (VIF) ranged from 1.229 to 1.557, indicating that the collinearity coefficients were deemed credible (See Table 1). In terms of educational resources, the pedestrian pathways were designed to connect kindergartens or daycares, providing convenient access to schools. Safety and the inclusion of interesting points were identified as two crucial factors for a successful pedestrian pathway. Regarding safety, it was ensured that the pedestrian pathways were designed to be safe, with walkability and appropriate gradients. Additionally, measures were taken to separate pedestrians from vehicular traffic, including the proper separation of sidewalks and streets using landscape buffers. On-street parking along the sidewalk and bike lanes provided a safety barrier between pedestrians and motor vehicles. This was specifically important for children’s safety because their walking patterns are less predictable and more spontaneous^27^. Play and recreation mean the pedestrian pathway links to CPOI for children.

**Table 1:** Coefficients.

Model	Unstandardized coefficients			Standardized coefficients	t	Sig	Collinearity statistics	
	B	Std. error	Beta				Tolerance	VIF
1	(Constant)	0.887	0.671		1.323	0.193		
	Age	− 0.006	0.152	− 0.005	− 0.042	0.967	0.814	1.229
	Education resources	0.382	0.143	0.347	2.677	0.010	0.642	1.557
	Safety	0.435	0.141	0.368	3.093	0.003	0.762	1.312
	Play and recreation	0.106	0.065	0.195	1.645	0.107	0.774	1.293

a. Dependent Variable: satisfaction.

#### Data preparation and weighting

Guidelines were formulated drawing from existing literature. The concept of CFC played a significant role in fostering the satisfaction of children within neighborhoods. This involved ensuring easy access to a safe and clean environment, essential amenities and services, educational opportunities, green spaces, and the opportunity for children to forge new friendships and engage in play within a secure setting^28,32^. These factors were succinctly summarized as follows.

1. Current pedestrian data: the planned pedestrian pathway network will make maximum use of the existing roads and lanes. This study obtained the distance grid data of the existing roads and lanes.
2. Land use: the pedestrian pathway was built on the built land and to avoid parks or rivers.
3. Residential data: represented the potential walking demand, which was translated into population density. The larger the value, the stronger the walking demand. And the pedestrian pathway would link the residential area with other points.
4. Barriers: the safety of pedestrian pathways for children necessitates their isolation from vehicular traffic. Firstly, the road should implement traffic calming measures, such as speed humps, traffic islands, raised crosswalks, and chicanes, to reduce vehicle speeds and create safer environments for child pedestrians. The second goal was to create and maintain pedestrian infrastructure that was especially tailored to meet the requirements of children pedestrians. This included constructing and maintaining sidewalks, crosswalks, pedestrian bridges, and underpasses. Ensure that these facilities were well-maintained, clearly marked, and accessible.
5. Children's point of interest (CPOI): mainly in the form of children’s point of interest data. When planning steps, pay attention to contacting children's points of interest.
6. Trees: these provided comfortable places for children. Trees offer shade, creating cool and pleasant environments where children can seek respite from the heat and enjoy outdoor activities. Moreover, the presence of trees contributes to improved air quality by filtering pollutants and releasing oxygen, creating a healthier and more refreshing environment for children to play and explore. The calming effect of nature, combined with the visual appeal of trees, promotes a sense of tranquility and peace, creating a welcoming atmosphere for children to relax, engage in imaginative play, or simply enjoy the beauty of nature. Furthermore, trees provide opportunities for interactive experiences, such as climbing, swinging from branches, or building treehouses, fostering physical activity, and enhancing children's motor skills and coordination.

The classification scores of various factors were shown in Table 2 in AHP processing. . The AHP is generally employed for expert scoring. It involves having four experts directly provide scoring judgments for the relative importance, creating a judgment matrix. Subsequently, the scores are consolidated to obtain the final judgment matrix. Finally, the weights for each factor are calculated based on this matrix. The consistency testing of Cronbach's Alpha is 0.774, indicating its reliability (See Table 3).

**Table 2:** Classification scores of Child Pedestrian-Friendly Pathways.

Impact factor	Data category	Cost-oriented
Land use	Unused land	256
	Park	128
	Education resource	32
	Residential area	16
	Commercial land	8
	Municipal	4
	Rivers	2
Trees	Trees	8
CPOI	CPOI	8
Distance from roads or lanes	0–20	256
	20–40	128
	40–60	64
	60–80	32
	80–100	16
	100–120	8
	120–140	4
	140–160	2
Barriers	Fencing	256
	Bollards	128
	Pedestrian gates	64
	Raised pedestrian crossings	32
	Sidewalks and footpaths	16
	Signalized crosswalks	8
	Reflective markings	4
	Warning signs	2
Heritage	Heritage building	8

**Table 3:** Reliability statistics.

Cronbach's Alpha	Cronbach's Alpha based on standardized items	N of items
0.774	0.795	3

The mutual importance of each element was determined by the fuzzy analytic hierarchy process and determined its weight, as shown in Table 4. Based on the raster data of the cost of each factor, the weight was combined by superposition analysis, the comprehensive cost was obtained from the comprehensive consideration. However, engineering, social, and population factors of road planning and construction cost.

**Table 4:** Influence factor matrix and weight of road planning.

Factor	Land Use	Trees	CPOI	Distance from road	Barriers	Heritage	weight
Land use	0.500	0.400	0.400	0.400	0.283	0.200	0.113
Trees	0.600	0.500	0.499	0.499	0.400	0.300	0.153
CPOI	0.600	0.501	0.500	0.499	0.400	0.300	0.153
Distance from road	0.600	0.501	0.501	0.500	0.400	0.300	0.153
Barriers	0.700	0.600	0.600	0.600	0.500	0.400	0.194
Heritage	0.800	0.700	0.700	0.700	0.600	0.500	0.234

#### Data analysis

**Figure 5:**
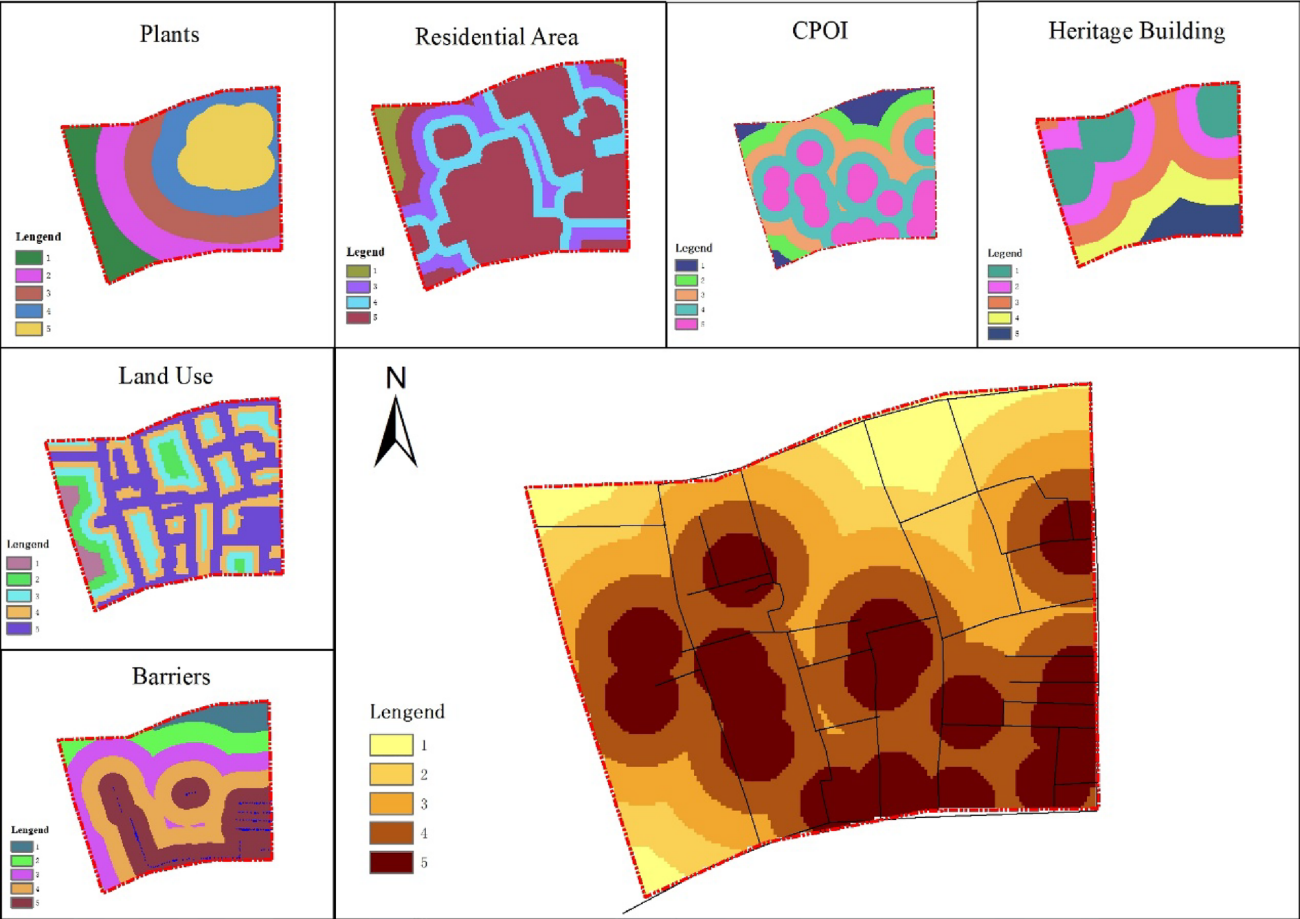
Data Analysis Result.

The developed GIS analysis model has been carefully packaged and consolidated, resulting in the creation of a highly functional process tool, as visually represented in Fig. 5. This tool served a crucial purpose by facilitating the efficient planning of a child-friendly pedestrian pathway. Notably, it offered the advantage of avoiding areas with high costs, such as water bodies and heritage buildings, thus minimizing potential financial burdens. Additionally, the tool optimized the utilization of existing CPOI, harnessing their potential benefits while simultaneously mitigating negative impacts on the environment. By incorporating these considerations, the tool contributed to a more sustainable and economically viable road planning process.

After conducting the spatial superposition analysis, the results were influenced by the existing lanes and roads. These lanes were able to be found on one or both sides of the roadway and serve to bridge gaps between important destinations within a community. In most plans, the pathways tended to rely more on the existing roads and overlook the importance of utilizing the lanes. However, it was worth noting that a pedestrian lane could serve as an interim or temporary pedestrian facility, particularly suitable for roads with low to moderate speeds and volumes (See Fig. 4).

#### Visualization and mapping

Based on the data analysis, the urban planner designed the safest pathway for children in the community. The pedestrian environment, consisting of paving and brick pavements, connected all CPOI and promotes children's growth and family comfort through appropriate design. The outcome included separate lanes for bicycles and pedestrians, aiming to contribute to Guangzhou becoming a more CFC.

### CFPP planning result

**Figure 6:**
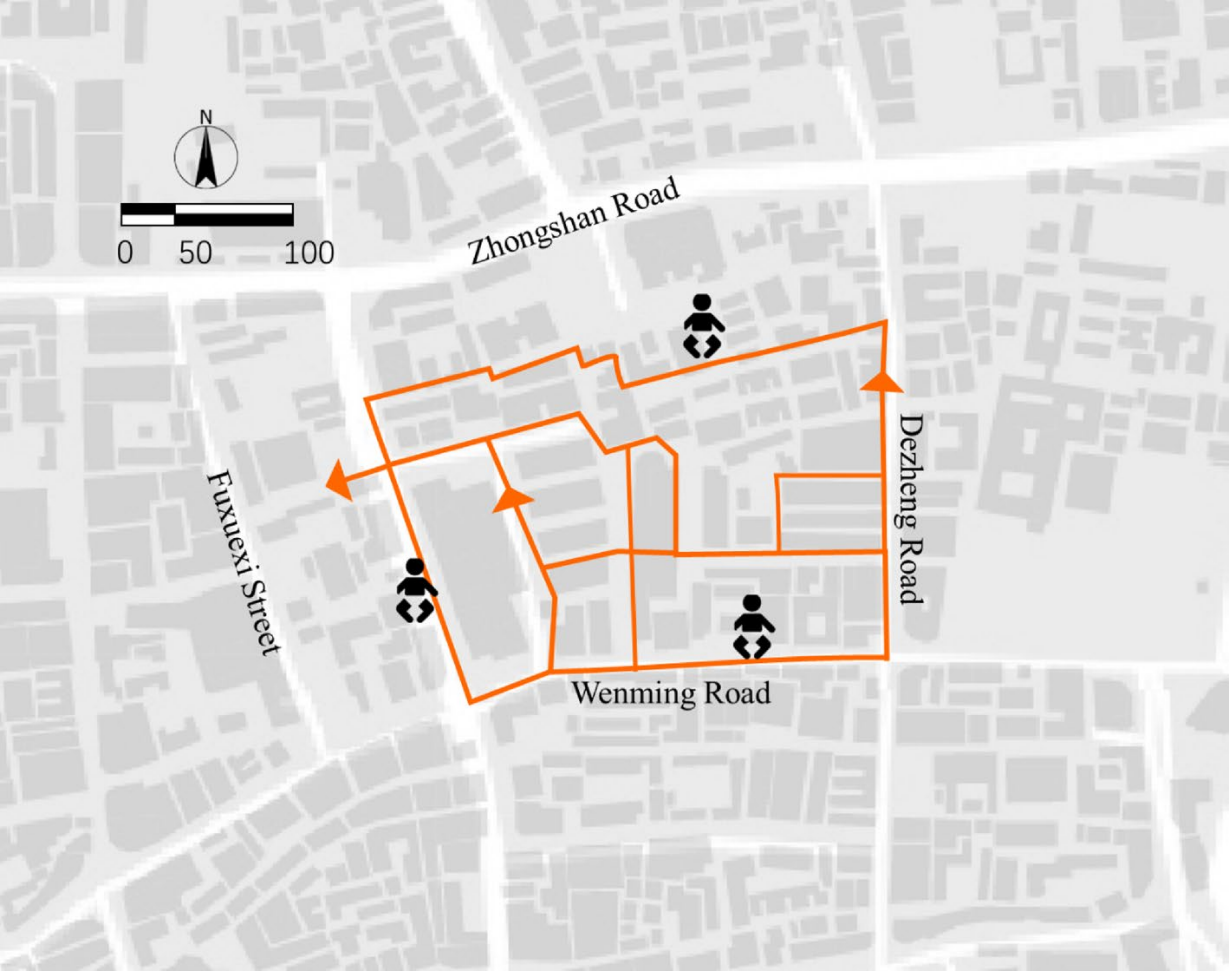
Walking routes proposed by the algorithm to meet the preferences of child (Legend: Map is made by ArcGIS 10.3.1 URL: https://www.arcgis.com/index.html).

The results of testing the algorithm's response to different combinations of user preferences are presented in Fig. 5. The algorithm was tested on a specific case using the settings illustrated in Fig. 6. This case aimed to identify a child-friendly pedestrian pathway.

**Figure 7:**
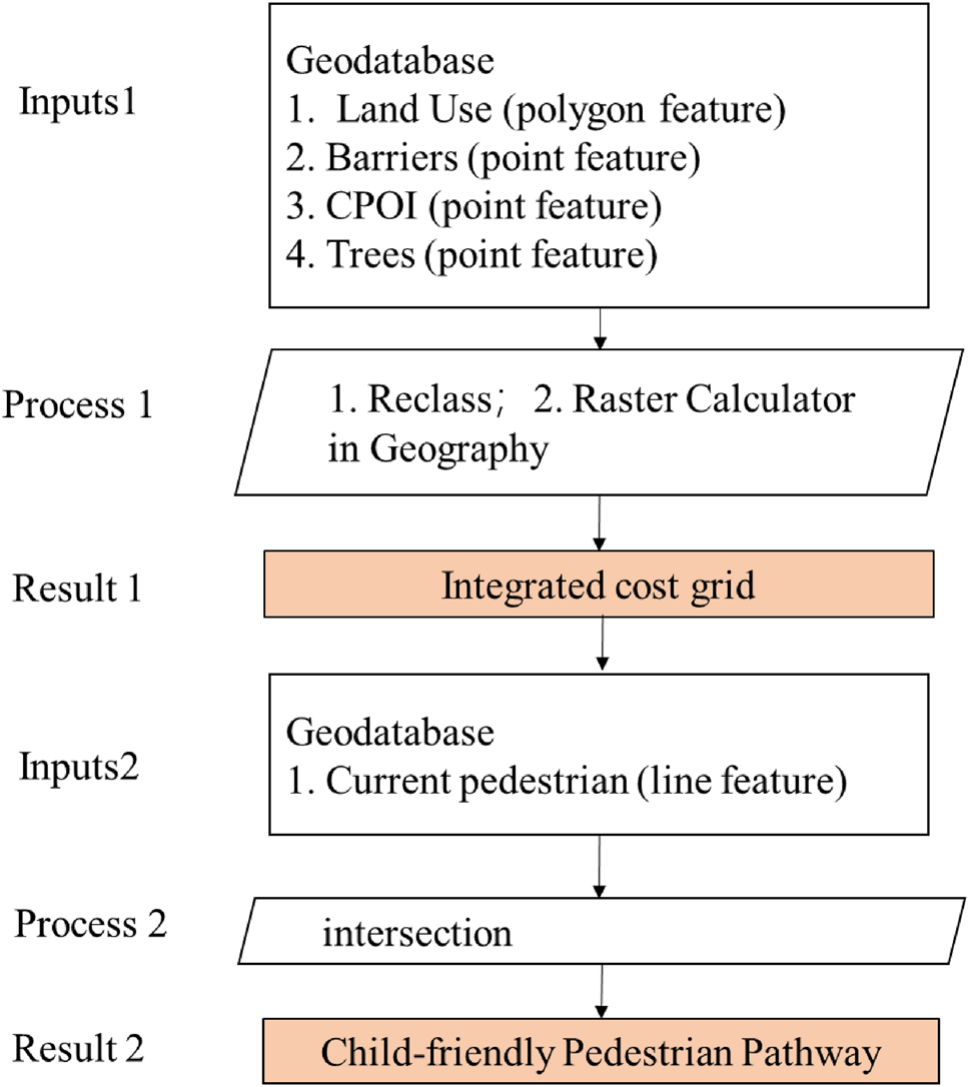
Flow chart of Child-Friendly Pathway intelligent planning based on GIS.

In the future, there are plans to develop an application that could be installed on mobile phones and similar devices, making it accessible to the general public. This application would enable cross-analysis between user behavior and the suggested routes generated by the algorithm. Additionally, user feedback regarding their satisfaction with the application could be used to validate the model (See Fig. 7).

### CFPP mechanism

Feedback mechanisms within the context of CFPP play a crucial role in engaging with children, parents, and communities. Social media platforms, particularly popular among older adolescents and young individuals with mobile connectivity, serve as a means to receive information, report incidents, and offer feedback or file complaints. Following the release of CFPP results, establishing feedback mechanisms becomes essential for collecting suggestions and ensuring continuous improvement of CFPP initiatives.

Efforts can be made to integrate consultations into existing programs and activities within established structures. It is advisable to centralize all gathered feedback into a dedicated database. A central database facilitates the tracking of feedback, documents actions taken to address complaints or suggestions, and provides analytical tools to discern trends in feedback. Viable database tools for this purpose include Excel, Microsoft Access, or an SPSS database.

## Conclusion and discussion

### Discussion

The study focused on two main objectives: first, to examine children's perceptions and meanings associated with a Child-Friendly City (CFC) and second, to explore the implications of these perceptions on children's interest in various spaces within their community. The findings highlighted the influence of the social milieu on the perceived child-friendliness and environmental friendliness of the community. Notably, Critical Points of Interest (CPOIs), spaces considered child-friendly, often emerged organically in inconspicuous places, demonstrating the importance of understanding the community from the perspective of its youngest members.

The innovative creation and application of an algorithm to automate the computation of child-friendly pedestrian routes (CFPP) was a major advancement in the study. This algorithm has proven to be an effective tool for managing large amounts of spatial data in Guangzhou, despite obstacles to user preferences. This methodology provides urban planners with a crucial tool for designing safe and child-friendly infrastructure, indicating a substantial development in intelligent planning methodologies.

The study emphasized the significance of creating child-friendly play areas along the CFPP, integrating basic utilities, and separating spaces based on the individual needs of different age groups. This insight provides a framework for more inclusive and personalized infrastructure in future urban planning and development efforts.

Moreover, the study highlighted the significance of ensuring safety, security, and the presence of adults within play spaces. These factors were identified as crucial elements contributing to the overall success of CFPPs in promoting active transportation for children. Child-friendly environments foster a sense of community and social integration among children by supporting their physical health, independence, and well-being.

### Policy implications

This study holds significant implications for urban planning policy, particularly concerning Child-Friendly Pedestrian Pathways (CFPP). Firstly, research findings can guide the selection of appropriate pathways, emphasizing regions aligned with children's views and preferences, fostering a sense of belonging and well-being. Secondly, develop and implement pathway guidelines, including child-friendly regulatory signs, enhancing visibility and appeal. Thirdly, implement protective measures, addressing damages promptly for ongoing safety and performance. Planning for CFPP involves considering suitable amenities, safety signage, and visual appeal. Implementation strategies include creating kid-friendly signs, ensuring access to facilities, and adding eye-catching elements. These policies underscore the importance of a thorough, child-centered approach for creating secure, accessible, and engaging urban spaces, ensuring a sustainable and child-friendly future as cities expand globally.

### Conclusion

The study offers a comprehensive examination of children's perceptions and meanings regarding Child-Friendly Cities (CFC). The development and successful implementation of an algorithm for Child-Friendly Pedestrian Pathways signify a significant advancement in intelligent planning methods, providing urban planners with an effective tool to prioritize children's safety and well-being.

It provides valuable insights into the significance of integrating child-friendly playgrounds alongside pedestrian walkways, emphasizing the need for specifically designed amenities catering to various age groups. The emphasis on security, safety, and adult presence in play areas underscores the necessity for implementing a comprehensive strategy to create spaces that genuinely meet the requirements of children.

However, this study acknowledges its limitations, notably its focus on a specific community, which restricts the generalizability of the results. Future studies should broaden the focus to encompass more cases to provide a more thorough understanding of the planning of child-friendly pedestrian pathways. Further research examining the qualitative features of Critical Points of Interest (CPOI) and possible losses and deteriorations in these areas offers a more nuanced perspective on the caliber of child-friendly settings.

## Data Availability

The datasets used and/or analysed during the current study available from the corresponding author on reasonable request.
